# Practitioners' Bookshelf

**Published:** 1892-07-09

**Authors:** 


### PRACTITIONERS' BOOKSHELF.

### MATERIA MEDICA.

It cannot be denied that in many points the Materia Medica* which Dr. Semple has compiled may be useful to students. We cannot say, however, that it supplies any generally felt want. Materia Medica, especially in its botanical aspect, is gradually being eliminated from the requirements of the examining boards of the colleges and universities, therapeutics and pharmaco-dynamics being largely substituted for the subjects of Materia Medica and pharmacy. A small text book, therefore, on the above subjects would be far more acceptable to the present-day student, than one on the subjects which Dr. Semple has chosen. In his preface the author remarks, "Many excellent treatises upon Materia Medica are in existence, but, with few exceptions, such as Bentley's 'Text book of Organic Materia Medica,' and Royle and Headland's 'Manual of Materia Medica and Therapeutics,' no illustrated volume has appeared since the publication of Pereira's 'Elements,' now some twenty years ago." This, however, appears to us no justification for collecting all the blocks which have been published in this barren period of twenty years, and for scattering them broadcast, no matter what their subject, through the pages of the work. There are 440 illustrations in about as many pages, but though as a picture-book it may appeal to the intellect of a child, it surely detracts from the dignity of a serious subject, that it should be treated in this puerile manner. We appeal to experts on the subject matter of the British Pharmacopœia to find justification for the following illustrations. A picture of a cow, another of a bull, two of pigs, five of sheep, one of a cock, two of sturgeon, two of codfish, one of a whale, and another ditto with icebergs. We hasten to satisfy the curiosity of the reader by informing him that the illustrations of the cow and the bull are supposed to lend interest to the otherwise dry subject of "lac bovis" (milk), and the five separate illustrations of the sheep are intended to amuse the student as he investigates the subject of wool fat or lanoline. The picture of the farm-yard cock, though surely the hen would have been more appropriate, illustrates the interesting subject of "Mistura spiritus vini gallici" (egg flip); and many other medicinal preparations are still more grotesquely illustrated. The subject matter of the book is not nearly so open to criticism; in fact, it is distinctly good, well up to date, and contains much useful information, especially in reference to the newer additions to the British Pharmacopœia, such as antifebrin, antipyrin, and magnesii sulphas effervescens. The latter is a compound containing Epsom salts, sugar, bicarbonate of sodium, tartaric acid, and citric acid. It is really a professional form of that highly esteemed quack preparation Eno's Fruit Salt. Dr. Semple has, however, made an exceedingly dry subject less dry than it is usually rendered, and possibly the 440 illustrations may divert the wandering thoughts of students into profitable channels.

* Elements of Materia Medica and Therapeutics, by Dr. Armand Semple, B.A.

